# A congenital portosystemic shunt in the neonatal period: case report and literature review

**DOI:** 10.3389/fped.2026.1836875

**Published:** 2026-07-15

**Authors:** Li Cheng, Shuwen Feng, Yin Shen, Xia Wang, Bingyan He, Dongchi Zhao, Pu Yang

**Affiliations:** 1 Department of Pediatrics, Women and Children's Hospital, Zhongnan Hospital of Wuhan University, Wuhan, China; 2 Department of Ultrasound Medicine, Zhongnan Hospital of Wuhan University, Wuhan, China

**Keywords:** Abernethy malformation, CPSS, EPSS, IPSS, neonate

## Abstract

A congenital portosystemic shunt (CPSS), a rare developmental malformation of the portal venous system, often presents with atypical clinical manifestations in the neonatal period that are prone to be missed or misdiagnosed. Herein, we report two neonatal CPSS cases. Case 1 initially manifested as respiratory distress, diffuse ecchymosis, and petechial hemorrhages and later caused progressive liver dysfunction, disseminated intravascular coagulation (DIC), and patent ductus arteriosus. Case 2 initially exhibited respiratory distress and multiple cutaneous hemangiomas, subsequently leading to progressive liver dysfunction, an increase in the number and size of hemangiomas, and severe pulmonary hypertension. Alongside our two neonatal CPSS cases, this article reviews 34 other neonatal cases reported in the literature over the past decade, aiming to enhance clinical recognition by summarizing the disease's clinical characteristics, diagnosis, treatment, and prognostic features during the neonatal period. Particularly, the early onset of idiopathic DIC, multiple cutaneous hemangiomas, progressive liver dysfunction, and pulmonary hypertension in neonates may be suggestive of CPSS. Furthermore, it underscores the critical importance of prenatal ultrasound screening, comprehensive multisystem assessment, and multidisciplinary care for the early diagnosis, individualized treatment, and improved prognosis of neonatal CPSS.

## 1 Introduction

A congenital portosystemic shunt (CPSS), a rare developmental malformation of the portal vein, is characterized by an abnormal communication between the portal venous system and the systemic venous system, resulting in the partial or complete diversion of portal venous blood away from the liver into the systemic circulation (1, 2). Its incidence is estimated at approximately 1/50,000–1/30,000 (3, 4). CPSS can be detected by a prenatal ultrasound or identified while evaluating a positive neonatal galactosemia screening, diagnosing and treating CPSS complications, or as an incidental finding on an abdominal ultrasound. However, the clinical manifestations of CPSS during the neonatal period are vague and non-specific, rendering early diagnosis a formidable challenge and causing them to frequently escape detection in the early postnatal period. With advances in imaging techniques and further recognition of CPSS, the number of reported cases has been increasing since its first description by Abernethy in 1793 (5). CPSS is primarily categorized into intrahepatic portosystemic shunts (IPSSs) and extrahepatic portosystemic shunts (EPSSs). The Park classification, the most widely adopted anatomical system for IPSS, subdivides these shunts into five types, wherein type V is designated as patent ductus venosus (PDV) (6). According to the Abernethy classification, EPSS is classified into two subtypes.

Clinical manifestations of CPSS in the neonatal period are variable, depending on the age of onset, shunt volume, and shunt type. Abnormal liver function, hyperammonemia, and hypoglycemia represent relatively common clinical manifestations. In addition, neonates with CPSS may present with intrauterine growth restriction (IUGR), respiratory distress, coagulation disorders, thrombocytopenia, or cholestasis. Moreover, CPSS is not only associated with congenital heart disease (CHD), Down syndrome, and urinary malformations but is also associated with various other conditions such as hepatopulmonary syndrome, pulmonary hypertension (PH), and liver tumors (7, 8).Current treatment for CPSS includes conservative management, endovascular embolization, surgical ligation, and liver transplantation (9). Although IPSS frequently close spontaneously before 2 years of age, EPSS may persist throughout life and potentially cause life-threatening complications. Consequently, early detection, systematic assessment, and appropriate intervention are paramount for achieving favorable outcomes in such patients.

The clinical manifestations in the neonatal period are highly variable. Therefore, in this study, we retrospectively analyzed two patients with neonatal CPSS admitted with disseminated intravascular coagulation (DIC) and multiple cutaneous hemangiomas, serving as the prominent clinical manifestations, and reviewed relevant literature on neonatal CPSS to investigate the clinical manifestations, diagnostic approaches, treatment strategies, and outcomes of neonatal CPSS, aiming to improve the early diagnosis and prognosis of the disease.

## 2 Case One

**Figure 1:**
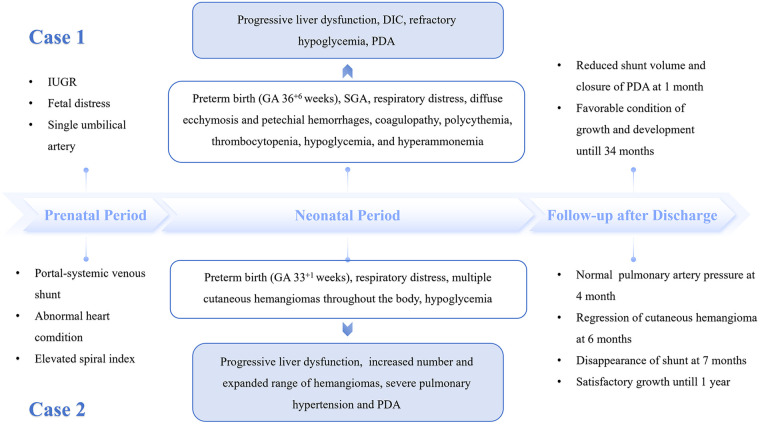
Episodes of two congenital portosystemic shunt cases. DIC, disseminated intravascular coagulopathy; GA, gestational age; IUGR, intrauterine growth restriction; PDA, patent ductus arteriosus; SGA, small for gestational age.

**Figure 2:**
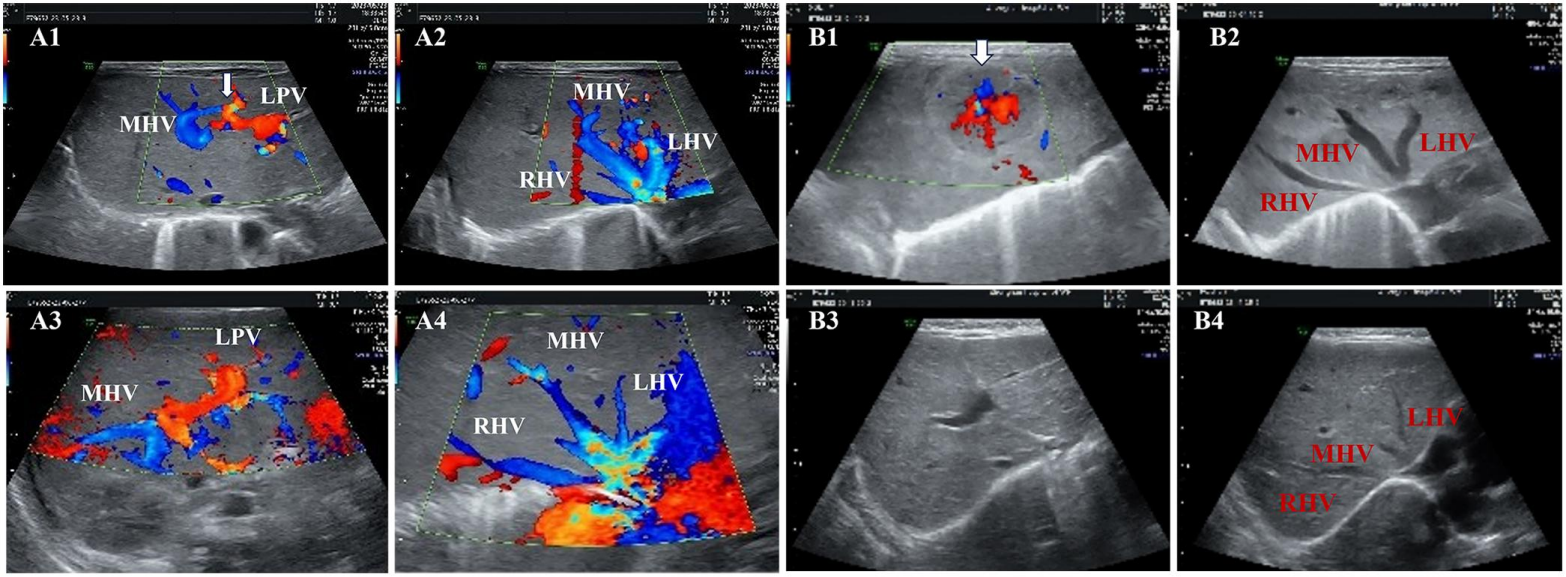
The hepatic ultrasound image of two congenital portosystemic shunt cases. **(A1–4)** Case 1—serial changes on hepatic ultrasound images. **(A1)** The arrow indicates the left portal vein branch with communication to the middle hepatic vein; **(A2)** hepatic vein dilatation secondary to the shunt (at admission); **(A3)** no visible communication between the portal vein and the hepatic vein (at 1-month follow-up); **(A4)** reduction in the caliber of the hepatic vein after the closure of the shunt. **(B1–4)** Case 2—serial changes on hepatic ultrasound images. **(B1)** The arrow indicates a portosystemic shunt; **(B2)** hepatic vein dilatation secondary to portosystemic shunting, and the degree of shunt flow is proportional to the number of hyperechoic foci (at admission); **(B3)** the hyperechoic foci resolved after the closure of the shunt (at 7-month follow-up); **(B4)** reduction in the caliber of the hepatic vein after the closure of the shunt. PV, portal vein; LPV, left portal vein; LHV, left hepatic vein; LRV, left renal vein; MHV, middle hepatic vein; RPV, right portal vein; RHV, right hepatic vein; SV, splenic vein; UV, umbilical vein.

A male infant, whose prenatal color Doppler ultrasonography (CDUS) demonstrated IUGR and a single umbilical artery, was delivered at a gestational age of 36^+6^ weeks via emergency cesarean section due to fetal distress. The infant had a birthweight of 2,190 g (below the 10th percentile), with Apgar scores of 8 at 1 min and 9 at 5 min. The primary symptoms after birth were respiratory distress, diffuse ecchymosis, and petechial hemorrhages across the body. Laboratory investigations showed abnormal coagulation [prolonged prothrombin time (PT) and activated partial thromboplastin time (APTT) and decreased fibrinogen], polycythemia, thrombocytopenia, hyperbilirubinemia, hypoglycemia, and hyperammonemia in the early postnatal period. As the disease progressed, the infant developed DIC within 24 h after birth (Table 1), refractory hypoglycemia, progressive liver dysfunction (transaminase elevation and cholestasis), and a cardiac murmur. A congenital portal-systemic venous shunt with hepatic vein and artery thickening was detected via an abdominal ultrasound within 48 h of birth. The liver magnetic resonance imaging (MRI) further revealed a thickening and tortuosity of the left portal vein branch with communication to the middle hepatic vein (IPSS II, single or multiple shunts connecting portal vein branches to the hepatic veins within a single liver lobe). Echocardiography showed a patent ductus arteriosus (PDA), a patent foramen ovale (PFO), and mild pulmonary valve stenosis. During hospitalization, non-invasive respiratory support was provided, as well as heparin and plasma therapy to regulate coagulation, glucocorticoids to promote platelet production, and hepatoprotective and choleretic measures for symptomatic management (Figure 1). The infant was discharged after 21 days of treatment, with a considerable improvement in clinical manifestations and laboratory indicators. The whole-exome sequencing (WES) ultimately revealed a pathogenic heterozygote in *WAC* [NM_016628.5: c.1810G>T (p. Glu604Ter)], which is not directly related to CPSS. At 1 month of age, a liver ultrasound revealed reduced shunt volume, whereas echocardiography indicated PDA closure (Table 1 and Figure 2). The child is currently 2 years and 10 months old with favorable growth and development.

**Table 1:** Progressive deterioration and subsequent improvement of laboratory features after treatment in two cases.

Case	Time (day of life)	TB/DB (µmol/L)	ALT (IU/L)	PT (s)	APTT (s)	DD (ng/mL)	FIB (mg/dL)	Ammonia (µmol/L)	Blood glucose (mmol/L)	PLT (×10^9^/L)	Echocardiography
1	D 1	/	/	27.6	>120	804	59	/	1.9	40	/
	D 2	191.2/23.4	22	25	93.7	264	64	/	2.0	16	PDA with a predominant left-to-right shunt
	D3	227/32	17	22.3	63	1,407	92	182	2.2	26	/
	D 5	182.2/42.4	35	17.5	57.4	5,396	59	144.2	4.8	42	/
	D 6	181.2/69.1	15	14.2	69	24,319	78	201.6	5.2	21	/
	D 8	188.8/86.1	33	12.8	68.3	13,246	103	/	4.9	39	PDA with a predominant left-to-right shunt; a mild pulmonary valve stenosis; PFO with a predominant left-to-right shunt
	D 10	172.2/88	42	13.1	59.9	756	142	181.4	5.0	79	/
	D13	113.4/59.5	56	13.8	44.4	698	133	155.3	5.4	176	/
	D15	76.8/37.9	52	16.6	65.7	460	159	187.6	5.3	145	mild pulmonary valve stenosis; ASD with a predominant left-to-right shunt
	D18	48.3/17.7	36	14.1	47.8	20,428	124.7	/	5.2	126	/
	D21	32.2/11	29	14.1	46.3	19,411	162	47.1	5.1	118	/
	D29	23.2/14.6	30	13.4	51.7	86	207	/	5.3	203	/
	D59	20.3/9.8	22	12.4	42.6	177	258	/	3.9	186	ASD with a predominant left-to-right shunt
	D 90	/	/	/	/	/	/	/	/	/	Normal
2	D1	26.2/9.4	3	13	62.1	782	138	60.7	1.6	247	Pulmonary hypertension (PASP∼53 mmHg); PDA with a predominant left-to-right shunt
	D 7	144.7/11.6	6	12.6	59.3	742	167	32.7	4.8	191	Pulmonary hypertension (PASP∼60 mmHg)
	D12	89.2/5.4	4	12.1	44.0	1,189	256	52.4	5.1	155	Pulmonary hypertension (PASP∼59 mmHg)
	D 19	93/9.3	5	11.7	45.3	NA	198	59.0	5.3	237	Pulmonary hypertension (PASP∼51 mmHg)
	D30	70.7/7.3	<10	/	/	/	/	/	/	260	Pulmonary hypertension (PASP∼43 mmHg)
	D60	36.7/6.8	12	11.8	35.5	374	166	81.42	/	277	Pulmonary hypertension (PASP∼40 mmHg)
	D 95	/	/	/	/	/	/	/	/	323	Pulmonary hypertension (PASP∼32 mmHg)
	D139	/	/	/	/	/	/	/	/	/	Normal

ALT, alanine aminotransferase; ASD, atrial septal defect; APTT, activated partial thromboplastin time; DB, direct bilirubin; DD, D-Dimer; FIB, fibrinogen; NA, not available; PDA, patent ductus arteriosus; PFO, patent foramen ovale; PLT, platelet count; PT, prothrombin time; TB, total bilirubin;/, not available.

## 3 Case Two

**Figure 3:**
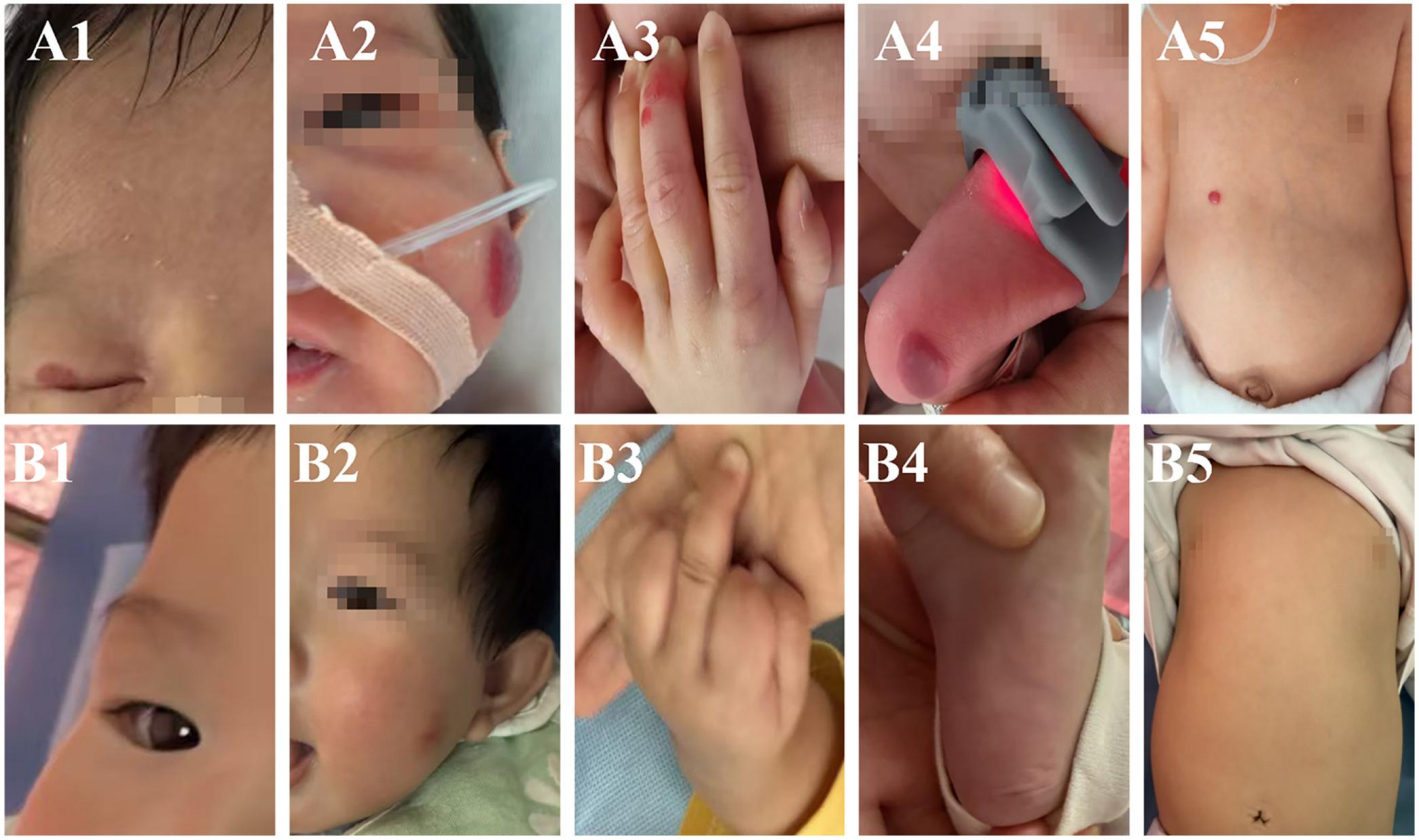
The change of the cutaneous hemangiomas in case 2. **(A1–A5)** The cutaneous hemangiomas in case 2 during hospitalization; **(B1–B5)** the cutaneous hemangiomas have become lighter or faded away at 7-month follow-up.

The male infant had a portal-systemic venous shunt, CHD, increased umbilical cord spiral index, and widened cerebellomedullary cistern as shown by a prenatal CDUS. The infant was born at a gestational age of 33^+1^ weeks via cesarean section, with a birthweight of 2,310 g and Apgar scores of 9 at 1 and 5 min. Following birth, the infant immediately exhibited respiratory distress, multiple cutaneous hemangiomas across the body, and concurrent hypoglycemia. A physical examination upon admission revealed various scattered erythematous macules: a macule on the left cheek (approximately 1 × 0.5 cm), a macule on the left forearm (approximately 0.5 × 0.2 cm), and scattered sesame-sized erythematous macules across the back. The infant presented with tachypnea, mild respiratory retraction, and a grade III/VI rumbling murmur in the precordial area. The infant also had hepatomegaly, with the liver edge located 4 cm below the costal margin. Laboratory tests were largely unremarkable, apart from hypoglycemia. A liver ultrasound revealed a thickening and tortuosity of the right portal vein branch with communication to the middle hepatic vein and right hepatic vein (IPSS II), slightly hyperechoic patchy areas, widened hepatic veins, and hepatomegaly. Echocardiography demonstrated a tubular PDA (0.53 cm in diameter), a PFO, and an enlarged right ventricle with a thickened wall. Additional findings included moderate tricuspid regurgitation and a widened pulmonary artery, accompanied by moderate PH, with an estimated systolic pressure of approximately 63 mmHg. Furthermore, the infant developed progressive liver dysfunction, and the number and size of hemangiomas increased (Figure 1). After admission, the infant underwent non-invasive respiratory support and received sildenafil for PH, oral propranolol for hemangiomas, and symptomatic management. Upon discharge within 22 days of comprehensive treatment, the infant's clinical manifestations and laboratory indicators showed significant improvement (Table 1). The infant continued receiving oral sildenafil and propranolol after leaving the hospital. At the 4-month follow-up, echocardiography revealed normalized pulmonary artery pressure, enabling the discontinuation of sildenafil. By 6 months of age, cutaneous hemangiomas had significantly diminished, prompting the cessation of propranolol (Figure 3). A liver ultrasound at 7 months confirmed the disappearance of the shunt (Figure 2). Currently followed up to 1 year, the infant demonstrates healthy growth and development.

## 4 Literature review

A structured literature search was conducted by two independent reviewers on 1 March 2026. The search strategy combined Medical Subject Headings and subheadings, including “congenital portosystemic shunt,” “CPSS,” “Congenital Intrahepatic Portosystemic Shunt,” “Congenital Extrahepatic Portosystemic Shunt,” “Abernethy malformation,”, “newborn,” “Infant,” and “neonate.” In addition, the synonyms, related indexing terms, and alternative spellings were also used. No language restrictions were applied, but reviews and conference abstracts were excluded. The databases searched included PubMed/MEDLINE, Embase, and SinoMed, covering the period from January 2015 to December 2025. These databases were selected for their comprehensive indexing of health-related literature, including primary studies. Thus, 168 articles were identified in the three databases. The PRISMA methodology was adopted to systematize study inclusion (10). The two researchers selected studies independently, with disagreements resolved by consensus. A staged analysis of the articles was performed by reading the titles and abstracts and then analyzing the full text of the articles for the final selection. PubMed was analyzed first, followed by Embase and SinoMed. The following exclusion criteria were used: repeated articles, type of article (Review/conference abstract), incomplete clinical data, and those who did not answer the research question (outside the theme). Full texts that met the eligibility criteria were also selected in a paired and independent way. The reasons for inclusion and exclusion are represented in Supplementary Figure 1.

This study analyzed clinical data from 36 neonatal CPSS cases, comprising the two cases presented above and additional 34 cases from the literature published over the past decade (11–30). The primary complications of neonatal CPSS encompassed transaminase elevation or hyperbilirubinemia (86.1%), hyperammonemia (52.8%), hypoglycemia (22.2%), thrombocytopenia or coagulation dysfunction (27.8%), CHD (33.3%), and IUGR or small for gestational age (25.0%) (Supplementary Appendix 1). These results closely align with those in domestic and international literature reports (12, 17, 30, 31), further verifying the multisystem involvement characteristic of neonatal CPSS.

Of all cases, eight children presented with chromosomal or genetic abnormalities; for example, three children had trisomy 21 syndrome (Supplementary Table 1). Although studies reported associations between chromosomal abnormalities (such as trisomy 21) and CPSS (20, 29, 32), the incidence and exact genetic mechanisms remain unclear.

In this study, 23 (63.9%) children with IPSS achieved favorable outcomes with conservative treatment alone, observing spontaneous shunt closure or a gradual reduction during the follow-up. Eleven children (30.5%), including four patients with EPSS, underwent interventional embolization or surgical ligation. The clinical outcomes demonstrated shunt closure or its significant reduction. The prognosis was generally positive for most children, with only two deaths recorded (5.6%) (Supplementary Table 1).

## 5 Discussion

CPSS, a rare developmental malformation of the portal venous system, is classified into intrahepatic and extrahepatic types based on the location of the shunt. Because of differences in shunt type and volume, the clinical manifestations in the neonatal period are highly variable, complicating early diagnosis. Thus, it is crucial to identify various distinct clinical manifestations for early diagnosis and intervention.

Although both patients with CPSS from our center were diagnosed with IPSS II, they exhibited markedly different clinical manifestations. Case 1 initially presented with hypoglycemia and diffuse ecchymosis, which was later confirmed to be DIC. In contrast, case 2 initially exhibited respiratory distress and multiple cutaneous hemangiomas, which significantly increased in number and size, accompanied by the development of severe PH.

These two cases in our center exhibited clinical features consistent with previously reported cases, including hyperammonemia and hypoglycemia, stemming from CPSS diverting portal venous blood directly into the systemic circulation and bypassing hepatic metabolism. In our center, case 1 experienced recurrent and unexplained episodes of hypoglycemia, aligning with findings from prior studies (28, 30). Nevertheless, our case experience and literature review reveal that hyperammonemia severity exhibits considerable variation even among patients sharing the same subtype. Although elevated blood ammonia levels typically signal significant portosystemic shunting, its concentration is modulated by multiple factors, including shunt volume, dietary composition, and intestinal function (22, 33).

In our case 1, severe coagulation dysfunction is the most prominent clinical feature. The infant presented with diffuse ecchymosis and petechial hemorrhages after birth, and primary laboratory investigations showed prolonged PT and APTT, decreased fibrinogen, markedly elevated D-dimer, and severe thrombocytopenia (platelet count nadir of 16 × 10⁹/L). DIC was diagnosed within 24 h based on the dynamic changes in the coagulation survey. Although positive CPSS results on a prenatal systematic ultrasound were not detected, given the presence of IUGR, rapid progression to DIC, and hepatic insufficiency in the early postnatal period, the first postnatal liver ultrasound screening was performed within 48 h after birth, and IPSS was identified. The reason for the coagulation dysfunction was that CPSS diminishes hepatic perfusion, impairing the liver's synthetic function and consequently reducing the production and activation of coagulation factors (3, 34). Furthermore, because thrombopoietin synthesis depends on portal blood flow (35), reduced portal perfusion caused by portosystemic shunting decreases intrahepatic thrombopoietin synthesis, thereby aggravating thrombocytopenia. In severe instances, such as case 1, this deterioration may progress to secondary DIC. As the shunt volume progressively decreased on subsequent ultrasound follow-up, both the DIC-related parameters (platelet count and coagulation function) and liver function showed marked improvement in case 1. Studies have shown that cats or dogs with CPSS exhibit diminished coagulation factor activity regardless of the shunt type. Surgical attenuation of the shunt leads to increased abnormalities in coagulation times and factors immediately after the surgery, yet these return to normal once the shunting has fully recovered (36, 37). We hypothesize that abnormal coagulation function is correlated with the magnitude of shunt flow, but its association with the shunt type remains unclear. Therefore, CPSS should be suspected when neonates present with unexplained severe DIC or refractory hypoglycemia.

In addition, growth restriction is highly prevalent in patients with CPSS, with up to 70% of affected neonates exhibiting impaired growth (38). In our center, case 1 exhibited IUGR. The association between CPSS and IUGR stems from reduced hepatic perfusion due to the shunt (39). Therefore, a dedicated sonographic evaluation of the umbilical–portal system in growth-restricted fetuses proves advantageous. In addition, a prenatal detection of shunts facilitates early consultation with a pediatric hepatologist and enables close postnatal follow-up.

Although CPSS is exceedingly rare, it is intrinsically linked to a constellation of congenital malformations (3, 7), and comorbidities significantly influence its prognosis. The most prevalent comorbidity is CHD, encompassing PDA, atrial septal defect (ASD), and ventricular septal defect (VSD) (40), which affect 17%–27% of individuals with CPSS (41). In case 1, echocardiography detected a PDA and ASD, whereas case 2 presented with PDA and PH. CHD is notably prevalent in patients with CPSS, yet the precise mechanism linking CPSS and CHD remains unclear. Some researchers argue that hemodynamic changes secondary to cardiac defects may potentially affect the persistence of vitelline veins during the development of the embryonic portal system, contributing to the development or persistence of CPSS (42). Moreover, PH causes a significant complication in these patients. A key concern involves the pathogenesis of PH associated with CPSS, specifically the hypothesis that vasoconstrictive substances, thrombosis, and high cardiac output elevate pulmonary artery pressure (8). In case 2, PH is likely associated with the PDA because heightened pulmonary blood flow from common left-to-right shunt lesions in CHD can evolve into pulmonary vascular remodeling and PH over time. CHD includes not only common defects such as PDA, ASD, and VSD but also complex anomalies such as heterotaxy, single ventricle, and inferior vena cava interruption. Failure to detect these may result in unexplained treatment failure (41). Hence, recognizing the clinical significance of the co-occurrence of these two conditions is essential. Furthermore, screening for CPSS in children with complex or unexplained CHD, along with a comprehensive assessment of cardiac function in children diagnosed with CPSS, is crucial for optimizing diagnostic and therapeutic strategies and improving patient prognosis.

Beyond the previously noted clinical manifestations and complications, case 2 was also remarkable for the presence of multiple cutaneous hemangiomas. Following treatment with oral propranolol, the cutaneous hemangioma significantly regressed. During the follow-up at 7 months of age, a liver ultrasound demonstrated the closure of the CPSS branches, and by 1 year of age currently, the infant continued to thrive with healthy growth and development. Although relatively uncommon, these cutaneous manifestations have been reported in approximately 5% of cases (43). The precise mechanism governing the relationship between hemangiomas and CPSS remains elusive. In this study, the patient with CPSS also had a cutaneous hemangioma and harbored an *EPHB4* mutation. *EPHB4* encodes a tyrosine kinase receptor involved in vascular development and remodeling, and its pathogenic variants are associated with capillary malformation-arteriovenous malformation syndrome 2, a distinct autosomal dominant disorder. Amyere et al. (44) confirmed that loss-of-function mutations in *EPHB4* underlie vascular malformation by disrupting the RAS-MAPK signaling pathway. Although the *EPHB4* gene alteration was detected in one patient with CPSS who presented with hemangioma, whether this variant is causally related to hemangioma development in patients with CPSS remains unclear. Therefore, hemangioma may serve as a clinical indicator of an underlying portosystemic shunt. However, the mechanism linking hemangiomas to CPSS remains unknown.

Reports of patients with CPSS are increasing, with trisomy 21 syndrome emerging as the most commonly observed genetic disorder associated with CPSS (45). However, the precise incidence and underlying genetic mechanisms are unclear. Although this study found three children exhibiting trisomy 21 syndrome, we found no chromosomal abnormalities among the patients from our center. Otherwise, during the diagnostic evaluation of case 1, WES identified a variant in *WAC*; however, the literature review showed that pathogenic heterozygous *WAC* variants are more closely associated with DeSanto–Shinawi syndrome (46); thus, we consider it as incidental background information.

Therefore, CPSS should be considered in children with multiple generalized cutaneous hemangiomas, IUGR, or trisomy 21 syndrome. A prompt liver ultrasound is essential to confirm the diagnosis and inform subsequent management.

CPSS is primarily diagnosed by imaging. An ultrasound can detect CPSS by directly visualizing the fistula or by observing indirect signs. These include the dilation of the umbilical vein and/or the IVC, along with non-visualization of the ductus venosus. In our results, case 2 and the other 12 (36.1%) children were prenatally diagnosed with portosystemic shunts by CDUS, whereas the remaining children were initially diagnosed via an abdominal ultrasound after birth. High-frequency prenatal CDUS monitoring is particularly crucial for prenatally diagnosed fetuses with CPSS or IUGR (47), which is of great significance for guiding perinatal management and improving prognosis. In addition, a dedicated hepatic sonography in neonates with the early onset of idiopathic DIC, multiple cutaneous hemangiomas, progressive liver dysfunction, and PH is recommended, which may contribute to the early detection of potential portal-system shunts. Meanwhile, prenatally diagnosing CPSS is essential to comprehensively evaluate the entire venous and cardiovascular system. For children with complex conditions necessitating surgical intervention, contrast-enhanced abdominal computed tomography/MRI and abdominal angiography offer invaluable insights into the classification and shunt volume assessment, with abdominal angiography standing as the undisputed gold standard for IPSS diagnosis (3).

Treatment strategies for CPSS encompass conservative non-invasive management, minimally invasive endovascular embolization, direct surgical ligation, and definitive liver transplantation (9). The primary objective of CPSS treatment is to arrest or reverse its systemic manifestations and forestall progression in patients who have surpassed the typical age window for spontaneous shunt closure (48, 49). Diagnosed before the age of 2 years, IPSS exhibits a significantly higher likelihood of spontaneous closure compared with EPSS. Currently, the optimal treatment regimen for children with CPSS is debated. Consequently, individualized plans are tailored according to the shunt type, shunt volume, and the severity of presenting complications. However, the primary controversy lies in distinguishing shunt-related symptoms and complications when patients develop severe issues or the shunt fails to close spontaneously. Some scholars advocate for asymptomatic children with anticipated spontaneous closure to undergo regular follow-up until the age of 2 years, deferring immediate surgery (49). Then, surgical intervention may be considered if the shunt persists beyond this age (50). For children experiencing significant shunt volume leading to complications such as PH, hepatopulmonary syndrome, hepatic tumor, or hepatic encephalopathy, particularly those with PDV, surgical closure of the abnormal shunt vessel is recommended to improve prognosis (6).

## 6 Conclusion

CPSS, typically presenting with multisystemic symptoms during the neonatal period, may be considered a potential diagnosis in infants exhibiting unexplained DIC, refractory hypoglycemia, IUGR, and multiple cutaneous hemangiomas. Given the low prenatal detection rate, a systematic prenatal ultrasound and a postnatal abdominal ultrasound screening urgently require enhancement. For most IPSS cases, conservative management remains the preferred approach owing to the high spontaneous closure rate. In contrast, cases involving PDV or EPSS often present with life-threatening complications, warranting interventional or surgical therapy.

## Data Availability

The original contributions presented in the study are included in the article/Supplementary Material, and further inquiries can be directed to the corresponding author.

## References

[1] Chaturvedi A Klionsky NB Saul D. Ultrasound with Doppler evaluation of congenital hepatic vascular shunts. Pediatr Radiol. (2018) 48(11):1658–71. 10.1007/s00247-018-4247-0 30194461

[2] Bellettini CV Wagner R Sette Balzanelo A de Souza Andretta AL Nascimento de Moura A Fabris CC. Congenital intrahepatic portosystemic shunt diagnosed during intrauterine life. Revista Paulista de Pediatria: Orgao Oficial da Sociedade de Pediatria de Sao Paulo. (2016) 34(3):384–7. 10.1016/j.rpped.2016.03.003 27133713 PMC5178127

[3] Bernard O Franchi-Abella S Branchereau S Pariente D Gauthier F Jacquemin E. Congenital portosystemic shunts in children: recognition, evaluation, and management. Semin Liver Dis. (2012) 32(4):273–87. 10.1055/s-0032-1329896 23397528

[4] Wang Y Yan Y Yang Z Wei J Liu G Pei Q. Prenatal diagnosis of congenital portosystemic shunt: a single-center study. J Obstet Gynaecol Res. (2020) 46(10):1988–93. 10.1111/jog.14403 32761766

[5] Abernethy J. Account of two instances of uncommon formation in the viscera of the human body: from the philosophical transactions of the royal society of London. Medical Facts and Observations. (1797) 7:100–8. 29106224 PMC5111139

[6] Zhilong Y Qimin C Lijun F Jun C Ming H Sheng C. Diagnosis and treatment of congenital portosystemic shunt in children. Chin J Pediatr Surg. (2023) 44(09):811–5. 10.3760/cma.j.cn421158-20220208-00075

[7] Francois B Lachaux A Gottrand F De Smet S. Prenatally diagnosed congenital portosystemic shunts. J Matern Fetal Neonatal Med. (2018) 31(10):1364–8. 10.1080/14767058.2017.1315093 28372492

[8] Uike K Nagata H Hirata Y Yamamura K Terashi E Matsuura T. Effective shunt closure for pulmonary hypertension and liver dysfunction in congenital portosystemic venous shunt. Pediatr Pulmonol. (2018) 53(4):505–11. 10.1002/ppul.23944 29359418

[9] Knirsch W Benz DC Bühr P Quandt D Weber R Kellenberger C. Catheter interventional treatment of congenital portosystemic venous shunts in childhood. Catheter Cardiovasc Interv. (2016) 87(7):1281–92. 10.1002/ccd.26362 26715199

[10] Page MJ McKenzie JE Bossuyt PM Boutron I Hoffmann TC Mulrow CD. The Prisma 2020 statement: an updated guideline for reporting systematic reviews. BMJ (Clinical Research ed). (2021) 372:n71. 10.1136/bmj.n71 33782057 PMC8005924

[11] Xue R Zhang J Wang Y Ding Y Xiang l Zhang L. A case report of neonatal congenital portosystemic shunts complicated with multiple hemangiomas and mutation of Ephb4 gene. Chinese Journal of Neonatology. (2023) 38(5):301–2. 10.3760/cma.j.issn.2096-2932.2023.05.009

[12] Mei J Tao X. Neonatal congenital intrahepatic portosystemic venous shunt with disseminated intravascular coagulation as the initial manifestation: 2 cases report and literature review. Chin J ObstetGynecol Pediatr. (2024) 20(03):322–30. 10.3877/cma.j.issn.1673-5250.2004.03.011

[13] Zhang J Li L. Surgical ligation of porto-systemic shunt for congenital extra-hepatic porto-systemic shunt: a report of two neonates. Chin J Pediatr Surg. (2024) 45(05):455–7. 10.3760/cma.j.cn.421158-20231102-00443

[14] Ifuku T Suzuki S Nagatomo Y Yokoyama R Yamamura Y Nakatani K. Congenital portosystemic venous shunt associated with 22q11.2 deletion syndrome: a case report. BMC Pediatr. (2022) 22(1):379. 10.1186/s12887-022-03447-3 35768799 PMC9245277

[15] Zhang JS Li L. Surgical ligation of a portosystemic shunt for the treatment of type ii Abernethy malformation in 12 children. J Vasc Surg Venous Lymphatic Disord. (2021) 9(2):444–51. 10.1016/j.jvsv.2020.08.001 32791304

[16] Xie E Zhang G Bu J. Neonatal congenital portosystemic shunt complicated with iliac artery-umbilical vein fistula: a case report and literature review. Chin J Neonatol. (2017) 32(04):287–90. 10.3760/cma.j.issn.2096-2932.2017.04.011

[17] Xu J Dai L Zhang J Wang J Liu Y. Congenital portosystemic shunts:2 cases report and review of literature. Chin J General Pract. (2023) 21(12):2173–6. 10.16766/j.cnki.issn.1674-4152.003316

[18] Kamali L Moradi M Ebrahimian S Masjedi Esfahani M Jafarpishe MS. Patent ductus venosus in an infant with direct hyperbilirubinemia. Clin Case Rep. (2019) 7(7):1430–4. 10.1002/ccr3.2266 31360505 PMC6637328

[19] Beard L Wymore E Fenton L Coughlin CR Weisfeld-Adams JD. Lethal neonatal hyperammonemia in severe ornithine transcarbamylase (OTC) deficiency compounded by large hepatic portosystemic shunt. J Inherit Metab Dis. (2017) 40(1):159–60. 10.1007/s10545-016-9985-2 27832417

[20] Yamaguchi H Kosugiyama K Honda S Tadao O Taketomi A Iwata S. Down syndrome with patent ductus Venosus and hepato-biliary-pancreatic abnormalities. Indian J Pediatr. (2016) 83(1):78–80. 10.1007/s12098-015-1797-0 26096864

[21] Chacko A Kock C Joshi JA Mitchell L Ahmad S. Patent ductus venosus presenting with cholestatic jaundice in an infant with successful trans-catheter closure using a vascular plug device. Indian J Radiol Imaging. (2016) 26(3):377–82. 10.4103/0971-3026.190419 27857466 PMC5036338

[22] Poeppelman RS Tobias JD. Patent ductus venosus and congenital heart disease: a case report and review. Cardiol Res. (2018) 9(5):330–3. 10.14740/cr777w 30344833 PMC6188041

[23] Van Houdt M van der Merwe J Gewillig M De Catte L. Prenatal 3d-ultrasound diagnosis of isolated intrahepatic portal-systemic shunt with intact ductus venosus: a case report and literature review. Radiol Case Rep. (2021) 16(5):1173–8. 10.1016/j.radcr.2021.02.037 33796163 PMC7995476

[24] Avula SK Verma S Ram A Lankala R. Rare cause of neonatal pulmonary hypertension: congenital intrahepatic portosystemic shunt through an aneurysm. Ann Pediatr Cardiol. (2021) 14(2):220–3. 10.4103/apc.APC_68_20 34103865 PMC8174622

[25] Gorsi U Kalra N Gupta P Rayasam K Thapa BR Bhagat H. Endovascular transjugular occlusion of congenital intrahepatic portosystemic venous shunt using simultaneous fluoroscopy and transabdominal ultrasound guidance: report of 2 cases. Curr Probl Diagn Radiol. (2020) 49(1):64–6. 10.1067/j.cpradiol.2018.03.007 29674011

[26] Kashgari A Al Otibi M. Congenital intrahepatic portosystemic venous shunt. Int J Pediatr Adolesc Med. (2020) 7(1):56–7. 10.1016/j.ijpam.2020.03.004 32373704 PMC7193070

[27] Plut D Gorjanc T. A case of a newborn with an intrahepatic congenital portosystemic venous shunt with concurrent congenital duodenal web. Acta Radiol Open. (2019) 8(6):2058460119854173. 10.1177/2058460119854173 31218081 PMC6563404

[28] Weigert A Bierwolf J Reutter H Gembruch U Woelfle J Ganschow R. Congenital intrahepatic portocaval shunts and hypoglycemia due to secondary hyperinsulinism: a case report and review of the literature. J Med Case Rep. (2018) 12(1):336. 10.1186/s13256-018-1881-y 30415638 PMC6231275

[29] Gong Y Zhu H Chen J Chen Q Ji M Pa M. Congenital portosystemic shunts with and without gastrointestinal bleeding—case series. Pediatr Radiol. (2015) 45(13):1964–71. 10.1007/s00247-015-3417-6 26209117

[30] Xu S Zhang P Hu L Zhou W Cheng G. Case report: clinical features of congenital portosystemic shunts in the neonatal period. Front Pediatr. (2021) 9:778791. 10.3389/fped.2021.778791 34926351 PMC8674941

[31] Cytter-Kuint R Slae M Kvyat K Shteyer E. Characterization and natural history of congenital intrahepatic portosystemic shunts. Eur J Pediatr. (2021) 180(6):1733–7. 10.1007/s00431-021-03949-9 33481107

[32] Tanya Chun P Chun T Files M Vo N McAdams RM. Percutaneous embolization of congenital portosystemic venous fistula in an infant with down syndrome. Case Rep Vasc Med. (2013) 2013:127023. 10.1155/2013/127023 24171135 PMC3792513

[33] Ponce-Dorrego MD Hernández-Cabrero T Garzón-Moll G. Endovascular treatment of congenital portosystemic shunt: a single-center prospective study. Pediatr Gastroenterol Hepatol Nutr. (2022) 25(2):147–62. 10.5223/pghn.2022.25.2.147 35360378 PMC8958053

[34] Murayama K Nagasaka H Tate K Ohsone Y Kanazawa M Kobayashi K. Significant correlations between the flow volume of patent ductus venosus and early neonatal liver function: possible involvement of patent ductus venosus in postnatal liver function. Arch Disease Childhood Fetal Neonatal Edition. (2006) 91(3):F175–9. 10.1136/adc.2005.079822 16449256 PMC2672699

[35] Sezai S Kamisaka K Ikegami F Usuki K Urabe A Tahara T. Regulation of hepatic thrombopoietin production by portal hemodynamics in liver cirrhosis. Am J Gastroenterol. (1998) 93(1):80–2. 10.1111/j.1572-0241.1998.080_c.x 9448180

[36] Kummeling A Teske E Rothuizen J Van Sluijs FJ. Coagulation profiles in dogs with congenital portosystemic shunts before and after surgical attenuation. J Vet Intern Med. (2006) 20(6):1319–26. 10.1892/0891-6640 17186844

[37] Tzounos CE Tivers MS Adamantos SE English K Rees AL Lipscomb VJ. Haematology and coagulation profiles in cats with congenital portosystemic shunts. J Feline Med Surg. (2017) 19(12):1290–6. 10.1177/1098612(17693490 29171354 PMC11104174

[38] Kivilevitch Z Kassif E Gilboa Y Weisbuch T Achiron R. The intra-hepatic umbilical-porto-systemic venous shunt and fetal growth. Prenat Diagn. (2021) 41(4):457–64. 10.1002/pd.5882 33340131

[39] Gorincour G Droullé P Guibaud L. Prenatal diagnosis of umbilicoportosystemic shunts: report of 11 cases and review of the literature. AJR Am J Roentgenol. (2005) 184(1):163–8. 10.2214/ajr.184.1.01840163 15615968

[40] Justino H. The significance of congenital portosystemic shunts in congenital heart disease and the bizarre phenomenon of alternating portosystemic and systemic-portal shunting. Interv Cardiol Clin. (2024) 13(3):307–18. 10.1016/j.iccl.2024.03.006 38839165

[41] Lambert V Ladarre D Fortas F Durand P Hervé P Gonzales E. Cardiovascular disorders in patients with congenital portosystemic shunts: 23 years of experience in a tertiary referral centre. Arch Cardiovasc Dis. (2021) 114(3):221–31. 10.1016/j.acvd.2020.10.003 33281106

[42] Papamichail M Pizanias M Heaton N. Congenital portosystemic venous shunt. Eur J Pediatr. (2018) 177(3):285–94. 10.1007/s00431-017-3058-x 29243189 PMC5816775

[43] Guérin F Franchi Abella S McLin V Ackermann O Girard M Cervoni JP. Congenital portosystemic shunts: vascular liver diseases: position papers from the francophone network for vascular liver diseases, the French association for the study of the liver (AFEF), and ERN-rare liver. Clin Res Hepatol Gastroenterol. (2020) 44(4):452–9. 10.1016/j.clinre.2020.03.004 32279979

[44] Amyere M Revencu N Helaers R Pairet E Baselga E Cordisco M. Germline loss-of-function mutations in EPHB4 cause a second form of capillary malformation-arteriovenous malformation (CM-AVM2) deregulating RAS-MAPK signaling. Circulation. (2017) 136(11):1037–48. 10.1161/circulationaha.116.026886 28687708

[45] Nohomovich B Nguyen MHN Fakhoury J Cameron RC Gomes T. Down syndrome patients with congenital portosystemic shunts: a case report and review. Case Rep Gastroenterol. (2023) 17(1):367–75. 10.1159/000535477 38111805 PMC10727516

[46] Ho S Luk HM Lo IFM. Extending the phenotype of Desanto-Shinawi syndrome: a case report and literature review. American Journal of Medical Genetics Part A. (2022) 188(3):984–90. 10.1002/ajmg.a.62571 34797027

[47] Francois B Gottrand F Lachaux A Boyer C Benoit B De Smet S. Outcome of intrahepatic portosystemic shunt diagnosed prenatally. Eur J Pediatr. (2017) 176(12):1613–8. 10.1007/s00431-017-3013-x 28913555

[48] Franchi-Abella S Gonzales E Ackermann O Branchereau S Pariente D Guérin F. Congenital portosystemic shunts: diagnosis and treatment. Abdominal Radiol (New York). (2018) 43(8):2023–36. 10.1007/s00261-018-1619-8 29730740

[49] McLin VA Franchi Abella S Debray D Guérin F Beghetti M Savale L. Congenital portosystemic shunts: current diagnosis and management. J Pediatr Gastroenterol Nutr. (2019) 68(5):615–22. 10.1097/mpg.0000000000002263 30628988

[50] Fahmy DM Mitchell PD Jonas MM. Presentation, management, and outcome of congenital portosystemic shunts in children: the Boston Children's Hospital experience. J Pediatr Gastroenterol Nutr. (2022) 75(1):81–7. 10.1097/mpg.0000000000003450 35442217

