# Supplementary material for: A congenital portosystemic shunt in the neonatal period: case report and literature review
Source: Front Pediatr. 2026 Jul 15;14:1836875. doi: 10.3389/fped.2026.1836875 (PMC13415552; doi:10.3389/fped.2026.1836875)

Supplementary Material

# Supplementary Table 1. The clinical characteristic, treatment, and outcome of 36 cases with CPSS in the neonatal period

| Case | Citation | GA  (week) | Age at diagnosis (day) | Gender | Clue for  investigation | Associated anomalies | Chromosomal or genetic abnormality | Type of shunt | Anatomy | Fetal US findings | Treatment | Outcome |
| --- | --- | --- | --- | --- | --- | --- | --- | --- | --- | --- | --- | --- |
| 1 | Cheng L  et al. | 36 | 1 | M | IUGR; DIC; Hyperammonemia; Thrombocytopenia;  Hypoglycemia;  Progressive liver dysfunction; | PDA;  Pulmonary stenosis | WAC gene variant NM_016628.5:c.1810  G>T(p.Glu604Ter) | IPSS II | LPV-MHV | Abnormity of prenatal US | Conservative | Age 1 months: decrease of shunt |
| 2 | Cheng L  et al. | 33 | 1 | M | Dyspnea; Hemangioma;  Hypoglycemia;  Hyperammonemia;  Progressive liver dysfunction; | PH | Chromosomal normal | IPSS II | RPV-MHV RHV | Abnormity of prenatal US | Conservative | Age 7 months: disappearance of shunt |
| 3 | Mei X  et al. (11) | 37 | 1 | M | Hemangioma; Dyspnea; | / | EPHB4 gene variant  Chr:7100416271  c.1298-5C>T | IPSS IV | LPV-LHV;  RPV-MHV | Abnormity of prenatal US | Interventional treatment | Age 30 months: disappearance of shunt |
| 4 | Juan M  et al. (12) | 36 | 1 | M | DIC; Progressive liver dysfunction; Thrombocytopenia | PH; PDA; | / | IPSS II | LPV-MHV | / | Conservative | Age 16 months: disappearance of shunt |
| 5 | Juan M  et al. (12) | 36 | 1 | M | DIC; Progressive liver dysfunction; Thrombocytopenia | PDA | Normal | IPSS  (PDV) | LPV-IVC | / | Conservative | Age 9 months: disappearance of shunt |
| 6 | Zhang J  et al. (13) | / | 2 | F | Jaundice;  Hyperammonemia;  Liver dysfunction | / | / | EPSS type II | PV–IVC | / | Surgical treatment | Age 68 months: disappearance of shunt |
| 7 | Zhang J  et al. (13) | / | 2 | F | Jaundice;  Liver dysfunction;  Hyperammonemia | / | / | EPSS type II | SV–LRV | / | Surgical treatment | Age 1 months: disappearance of shunt |
| 8 | Ifuku T  et al. (14) | 35 | 1 | M | Hyperammonemia;  Liver dysfunction; | CHD; hyper-galactosemia;  characteristic facial features | 22q11.2 deletion | EPSS type II | SV–LRV | / | Interventional treatment | Age 3 months: disappearance of shunt |
| 9 | Zhang J  et al. (15) | / | 10 | F | Jaundice;  Liver dysfunction | / | / | EPSS type II | PV–IVC | Abnormity of prenatal US | Surgical treatment | Disappearance of shunt |
| 10 | Xie E  et al. (16) | 39 | 4 | M | Dyspnea; | PH; PDV | / | IPSS  (PDV) | PV-UV | / | / | Death |
| 11 | Xu J  et al. (17) | 40 | 12 | M | Dyspnea; Jaundice;  Hyperammonemia;  SGA;  Thrombocytopenia | PDA | / | IPSS II | PV-RHV | / | Conservative | Age 2 months: disappearance of shunt |
| 12 | Xu J  et al. (17) | / | 1 | F | / | ASD | / | IPSS II | LPV-MHV  RPV-MHV | Abnormity of prenatal US | Conservative | Age 3 months: disappearance of shunt |
| 13 | Kamali L  et al. (18) | 39 | 25 | M | Jaundice; Liver dysfunction | PDV | / | IPSS  (PDV) | LPV-LHV | / | Interventional treatment | Age 6 months: disappearance of shunt |
| 14 | Beard L  et al. (19) | / | 2 | M | Dyspnea; Lethargy; Seizures; Hyperammonemia | / | OTC gene deletion involving Xp21.1–Xp11.4 | IPSS II | LPV-LHV | / | / | Death |
| 15 | Yamaguchi H et al. (20) | 39 | 1 | F | DIC; Liver dysfunction; Thrombocytopenia | PDV; Down syndrome | Down syndrome | IPSS  (PDV) | PV-IVC | / | Conservative | Age 8 months: decrease of shunt |
| 16 | Chacko A et al. (21) | 38 | 21 | F | Jaundice;  Liver dysfunction | PDV | / | IPSS  (PDV) | PV-IVC | / | Interventional treatment | Age 1.5 months disappearance of shunt |
| 17 | Poeppelman RS et al. (22) | / | 2 | M | Liver dysfunction; Coagulation disturbance;  Hyperammonemia; Hypoglycemia | VSD; PDA; PDV | / | IPSS  (PDV) | PV-IVC | / | Conservative | Age 1.5 months disappearance of shunt |
| 18 | Van Houdt M et al. (23) | 37 | 1 | F | Hyperammonemia; IUGR | PDV | / | IPSS  (PDV) | PV-HV multiple | Abnormity of prenatal US | Conservative | Age 6 months: decrease of shunt |
| 19 | Avula SK et al. (24) | 35 | 1 | M | Liver dysfunction; Hyperammonemia;  Dyspnea; | PH | / | IPSS III | PV-HV multiple | / | Interventional treatment | Age 6months disappearance of shunt |
| 20 | Gorsi U et al. (25) | / | 3 | M | Dyspnea; Jaundice; Hyperammonemia; Hypoglycemia | / | / | IPSS I | LPV-IVC | / | Interventional treatment | Disappearance of shunt |
| 21 | Gorsi U et al. (25) | / | 2 | M | Jaundice; Liver dysfunction;  Hyperammonemia | / | / | IPSS II | RPV-RHV | / | Interventional treatment | Disappearance of shunt |
| 22 | Kashgari A et al. (26) | 38 | 7 | F | Liver dysfunction; vomiting and poor feeding | / | / | IPSS II | LPV-LHV | / | Conservative | Age 3 months: decrease of shunt |
| 23 | Plut D  et al. (27) | 39 | 1 | M | IUGR; Vomiting and poor feeding; Jaundice; Liver dysfunction; Hyperammonemia | / | / | IPSS II | LPV-LHV | / | Conservative | Age 6 months disappearance of shunt |
| 24 | Weigert A et al. (28) | / | 1 | M | Jaundice; Liver dysfunction; Hypoglycemia | non-compaction  cardiomyopathy | / | IPSS II | LPV-MHV | / | Interventional treatment | Age 3 months disappearance of shunt |
| 25 | Gong Y  et al. (29) | / | 11 | M | Jaundice; Thrombocytopenia | Down syndrome | Down syndrome | IPSS I | RPV-IVC | / | Conservative | Decrease of shunt |
| 26 | Gong Y  et al. (29) | / | 5 | F | IUGR; Jaundice | PDA; | / | IPSS II | LPV-MHV | / | Conservative | Decrease of shunt |
| 27 | Xu S  et al. (30) | 38 | 19 | M | Jaundice; Thrombocytopenia | PDA; Down syndrome; ASD | Down syndrome | IPSS I | LPV-IVC | / | Conservative | Age 8 months: shunt present on US |
| 28 | Xu S  et al. (30) | 37 | 1 | M | IUGR; Jaundice | / | / | IPSS II | LPV-MHV | Abnormity of prenatal US | Conservative | Age 10 months: disappearance of shunt |
| 29 | Xu S  et al. (30) | 39 | 13 | M | Jaundice; Hyperammonemia | Hypospadias | / | IPSS  (PDV) | LPV-LHV | / | Conservative | Age 13 months: disappearance of shunt |
| 30 | Xu S  et al. (30) | 38 | 7 | M | Jaundice; Hypoglycemia;  Hyperammonemia | / | / | IPSS II | LPV-MHV | / | Conservative | Age 36 months: disappearance of shunt |
| 31 | Xu S  et al. (30) | 37 | 7 | M | Hepatomegaly, hydropericardium; Hyperammonemia;  Liver dysfunction; | Noonan syndrome-8, pulmonary stenosis, ASD, PDA, hypertrophic cardiomyopathy | Noonan syndrome-8 | IPSS I | LPV-IVC | Abnormity of prenatal US; | Conservative | Age 12 months: disappearance of shunt |
| 32 | Xu S  et al. (30) | 39 | 6 | F | IUGR; Thrombocytopenia;  Hypoglycemia;  Liver dysfunction; | PDA | / | IPSS II | LPV-RHV, multiple | / | Conservative | Age 6 months: disappearance of shunt |
| 33 | Xu S  et al. (30) | 35 | 1 | M | Jaundice;  Hyperammonemia | / | / | IPSS  (PDV) | LPV-MHV | Abnormity of prenatal US | Conservative | Age 13 months: disappearance of shunt |
| 34 | Xu S  et al. (30) | 37 | 9 | M | Jaundice; IUGR; Hyperammonemia | / | / | IPSS  (PDV) | LPV-MHV | / | Conservative | Age 3 months: disappearance of shunt |
| 35 | Xu S  et al. (30) | 37 | 1 | M | IUGR; Hyperammonemia; Liver dysfunction; | ASD; metabolic osteopathy | / | IPSS II | LPV-LHV, MHV | Abnormity of prenatal US | Conservative | Age 4 months: disappearance of shunt |
| 36 | Xu S  et al. (30) | 38 | 20 | F | Jaundice; Thrombocytopenia;  Hypoglycemia; Hyperammonemia | / | / | IPSS II | LPV-LHV, multiple | / | Conservative | Age 4 months: disappearance of shunt |

ALT, alanine aminotransferase; ASD, atrial septal defect; CPSS, congenital portosystemic shunts; CHD, congenital heart disease; DB, direct bilirubin; DIC, disseminated intravascular coagulopathy; EPSS, extrahepatic portosystemic shunts; HPV, hepatic portal vein; IPSS, intrahepatic portosystemic shunts; IVC, inferior vena cava; IUGR, intrauterine growth restriction; F, female; M, male; /, not available; GGT, glutamyl transpeptidase; PDA, patent ductus arteriosus; PH, pulmonary hypertension; PDV, patent ductus venosus; PLT, platelet count; PT, prothrombin time; PV, portal vein; LPV, left portal vein; LHV, left hepatic vein; LRV, left renal vein; MHV, middle hepatic vein; RPV, right portal vein; RHV, right hepatic vein; SV, splenic vein; SGA, small for gestational age​; UV, umbilical vein; TB, total bilirubin

# Supplementary figure 1. The diagram of the publication retrieval and screening process


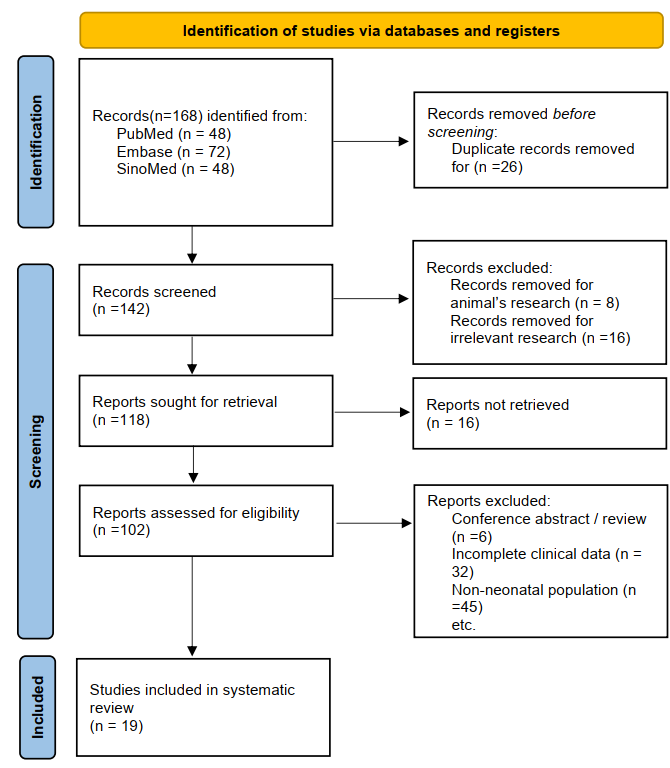

Supplement: Supplementary file 1 [file Supplementaryfile1.docx]
